# Patient Characteristics, Treatment and Outcome in Non-Ischemic vs. Ischemic Cardiogenic Shock

**DOI:** 10.3390/jcm9040931

**Published:** 2020-03-28

**Authors:** Benedikt Schrage, Jessica Weimann, Salim Dabboura, Isabell Yan, Rafel Hilal, Peter Moritz Becher, Moritz Seiffert, Alexander M. Bernhardt, Stefan Kluge, Hermann Reichenspurner, Stefan Blankenberg, Dirk Westermann

**Affiliations:** 1 Department of Cardiology, University Heart and Vascular Centre Hamburg, 20251 Hamburg, Germany; b.schrage@uke.de (B.S.); j.weimann@uke.de (J.W.); i.yan@uke.de (I.Y.); hilalrafel@hotmail.de (R.H.); m.becher@uke.de (P.M.B.); m.seiffert@uke.de (M.S.); s.blankenberg@uke.de (S.B.); 2 German Centre for Cardiovascular Research (DZHK), Partner Site Hamburg/Lübeck/Kiel, 20246 Hamburg, Germany; s.dabboura@uke.de; 3 Department of Cardiovascular Surgery, University Heart and Vascular Centre Hamburg, 20251 Hamburg, Germany; al.bernhardt@uke.de (A.M.B.); reichenspurner@uke.de (H.R.); 4 Department of Intensive Care Medicine, University Clinic Hamburg-Eppendorf, 20251 Hamburg, Germany; s.kluge@uke.de

**Keywords:** cardiogenic shock, non-ischemic, mechanical circulatory support

## Abstract

Aim: Evidence on non-ischemic cardiogenic shock (CS) is scarce. The aim of this study was to investigate differences in patient characteristics, use of treatments and outcomes in patients with non-ischemic vs. ischemic CS. Methods: Patients with CS admitted between October 2009 and October 2017 were identified and stratified as non-ischemic/ischemic CS based on the absence/presence of acute myocardial infarction. Logistic/Cox regression models were fitted to investigate the association between non-ischemic CS and patient characteristics, use of treatments and 30-day in-hospital mortality. Results: A total of 978 patients were enrolled in this study; median age was 70 (interquartile range 58, 79) years and 70% were male. Of these, 505 patients (52%) had non-ischemic CS. Patients with non-ischemic CS were more likely to be younger and female; were less likely to be active smokers, to have diabetes or decreased renal function, but more likely to have a history of myocardial infarction; and they were more likely to present with unfavorable hemodynamics and with mechanical ventilation. Regarding treatments, patients with non-ischemic CS were more likely to be treated with catecholamines, but less likely to be treated with extracorporeal membrane oxygenation or percutaneous left-ventricular assist devices. After adjustment for multiple relevant confounders, non-ischemic CS was associated with a significant increase in the risk of 30-day in-hospital mortality (hazard ratio 1.14, 95% confidence interval 1.04–1.24, *p* < 0.01). Conclusion: In this large study, non-ischemic CS accounted for more than 50% of all CS cases. Non-ischemic CS was not only associated with relevant differences in patient characteristics and use of treatments, but also with a worse prognosis. These findings highlight the need for effective treatment strategies for patients with non-ischemic CS.

## 1. Introduction

Cardiogenic shock (CS) originates in a state of severely reduced cardiac output, which leads to impaired end-organ perfusion and ultimately multi-organ failure [1]. CS occurs in up to 3% of patients with acute myocardial infarction, and, contrary to other cardiovascular diseases, there has not been much improvement regarding overall survival for patients with CS in the past decades [2]. Aside from early revascularization for patients with ischemic CS (e.g., CS due to acute myocardial infarction), there is no treatment which has shown to improve outcomes in CS [3,4]. Importantly, research on CS is focused on patients with ischemic CS, and past as well as ongoing randomized controlled outcome trials in this area used “CS due to ischemic origin” as a major enrolment criterion [5,6,7,8]. However, recent studies have indicated that only a minority of CS cases are explained by acute myocardial infarction, whereas the majority were non-ischemic CS cases, caused by, e.g., decompensated heart failure [9,10,11].

The marked differences in underlying pathology in ischemic vs. non-ischemic cases are likely to have an impact on diagnosis and management of CS. First of all, patient characteristics might differ in patients with ischemic vs. non-ischemic CS. This would have direct implications for diagnostic algorithms, which might have to account for different CS sub-types. Both sub-types demand different treatments. Ischemic CS originates from myocardial damage due to a mismatch in oxygen supply and demand. Early revascularization can resolve the mismatch and has shown to improve outcomes^3^. Additionally, if the patient is already in a state of ongoing CS, mechanical circulatory support devices might help to support cardiac output until the damaged heart recovers, although there is yet no randomized controlled trial which supports this approach [1,12]. On the contrary, non-ischemic CS can be caused by a variety of diseases, leading to severe myocardial dysfunction, such as acute heart failure (either as an acute decompensation in a chronic heart failure patient or as a de novo event) [11]. Unfortunately, there is as yet no evidence-based therapy targeting the severe myocardial dysfunction which can be applied in non-ischemic CS [2]. This limits the available options in the management of non-ischemic CS. The only remaining option currently is to bridge the patient until stabilization of native cardiac function or until heart transplantation or durable mechanical circulatory support implantation [2].

Currently, it is not well known if patients with non-ischemic CS present differently to the hospital as compared to patients with ischemic CS. Importantly, this would have implications for diagnostic algorithms in this field, as these might need to address relevant differences between both groups. Furthermore, there is a gap in the evidence regarding the use of therapies and overall risk in patients with non-ischemic vs. ischemic CS. However, more granular knowledge on these topics could help to optimize therapeutic management in these sub-populations, e.g., addressing a higher risk in one of the sub-populations with earlier/more aggressive treatment.

The aim of this study was to foster understanding of patient characteristics, use of treatments and outcomes in ischemic vs. non-ischemic CS, which might have implications for diagnostic and therapeutic management of CS in clinical practice.

## 2. Methods

### 2.1. Study Population

Consecutive patients with CS, identified via the ICD-10 code R.57 from the claims database of the University Heart and Vasculature Centre Hamburg, and aged ≥18 years, treated between 1 October 2009 and 31 October 2017, were retrospectively assessed. Digital case files were manually reviewed to ascertain that CS was indeed the primary diagnosis for hospital treatment, and patients not meeting this criterion were excluded (e.g., patients with secondary CS due to severe bleeding/trauma were excluded). For all included patients, baseline characteristics and in-hospital follow-up was available.

Patients were then stratified as having ischemic vs. non-ischemic CS based on the manual review of the digital case files. Hereby, ischemic CS was adjudicated if acute myocardial infarction, either with or without ST-segment elevation, was the main reason for CS/admission; non-ischemic CS was adjudicated in all other cases.

### 2.2. Statistical Analysis

Continuous variables are shown as medians (interquartile range) and compared using the Mann–Whitney U-test; binary variables are shown as counts (frequencies) and compared using the χ^2^ test.

Missing data was handled by chained-equation multiple imputation (50 imputed data sets; *R* package *mice*). Except creatinine kinase and high-sensitive troponin T, all the variables shown in Table 1, as well as use of catecholamines, vasopressors, extracorporeal membrane oxygenation and perivascular left-ventricular assist devices, were used for the multiple imputation.

**Figure 1:**
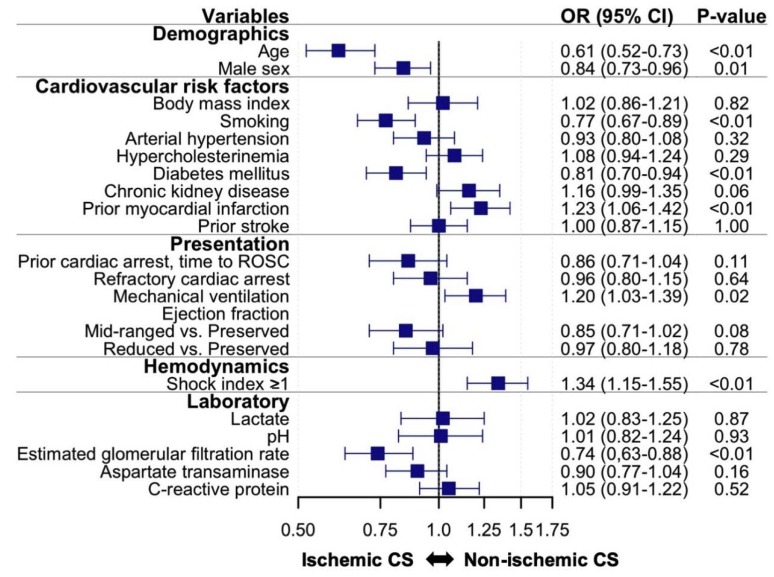
Patient characteristics of patients with non-ischemic vs. ischemic cardiogenic shock. Multivariable-adjusted logistic regression model with cause of cardiogenic shock (CS, non-ischemic vs. ischemic) as the dependent variable and all shown baseline characteristics as independent variables. Odds ratios (OR) are shown on the x-axis. Change in continuous variables was modeled as change per standard deviation. CI: confidence interval; ROSC: return of spontaneous circulation.

To assess differences in patient characteristics between patients with ischemic vs. non-ischemic CS, a logistic regression model with cause of CS as the dependent variable and selected patient characteristics as independent variables was fitted (Figure 1).

To assess differences in treatment between patients with ischemic vs. non-ischemic CS, logistic regression models with selected treatments (use of catecholamines, vasopressors, veno-arterial extracorporeal membrane oxygenation (ECMO) or perivascular left-ventricular assist devices (pLVAD)) as the dependent variables and the cause of CS as the independent variable were fitted. Additionally, these logistic regression models were also adjusted for age, sex, prior cardiac arrest, time to return of spontaneous circulation, shock index ≥1 (heart rate divided by systolic blood pressure), baseline lactate and baseline pH. Importantly, the decision to use or not use these devices was made by the treating physician at the given time, based on the respective case, and was not specified by an institutional protocol.

To assess differences in outcome between patients with ischemic vs. non-ischemic CS, the primary endpoint of 30 day in-hospital mortality was analyzed, whereas patients were censored upon discharge from the hospital. Survival probabilities of patients with ischemic vs. non-ischemic CS were estimated using the Kaplan–Meier method. To assess the association of cause of CS with the primary endpoint, a Cox regression model was fitted, adjusted for age, sex, prior cardiac arrest, time to return of spontaneous circulation, shock index ≥1, baseline lactate and baseline pH.

For all regression models, change in continuous variables was modeled as change per standard deviation. A *p*-value of <0.05 was considered statistically significant. All analyses were performed with R statistical software version 3.6.0 (R Foundation for Statistical Computing, Vienna, Austria).

### 2.3. Ethics

This project was performed in accordance with the Declaration of Helsinki and was approved by the local ethics committee.

## 3. Results

In total, 978 patients were included in this study. Of these patients, 505 patients (51.6%) were stratified as non-ischemic CS and 473 patients (48.4%) were stratified as ischemic CS. The mean age of the overall study cohort was 70 (interquartile range 58–79) years and 683 patients (69.8%) were males. A total of 567 patients (58.0%) had a prior cardiac arrest and 116 patients (28.0%) presented in refractory cardiac arrest. At baseline, lactate was 4.0 (interquartile range 2.0–8.0) mmol/L and pH was 7.3 (interquartile range 7.2–7.4). Detailed baseline characteristics are shown in Table 1.

### 3.1. Patient Characteristics

Baseline characteristics in Table 1 are unadjusted. Therefore, we performed multivariable-adjusted logistic regression to identify patient characteristics independently associated with ischemic vs. non-ischemic CS (Figure 1). Among the patient characteristics considered in our analyses, prior myocardial infarction, mechanical ventilation upon presentation and higher shock index were independently associated with higher odds of having non-ischemic CS. Conversely, higher age, male sex, active smoking, diabetes mellitus, and higher estimated glomerular filtration rate were independently associated with lower odds of having non-ischemic CS (e.g., higher odds of having ischemic CS).

### 3.2. Treatments

**Figure 2:**
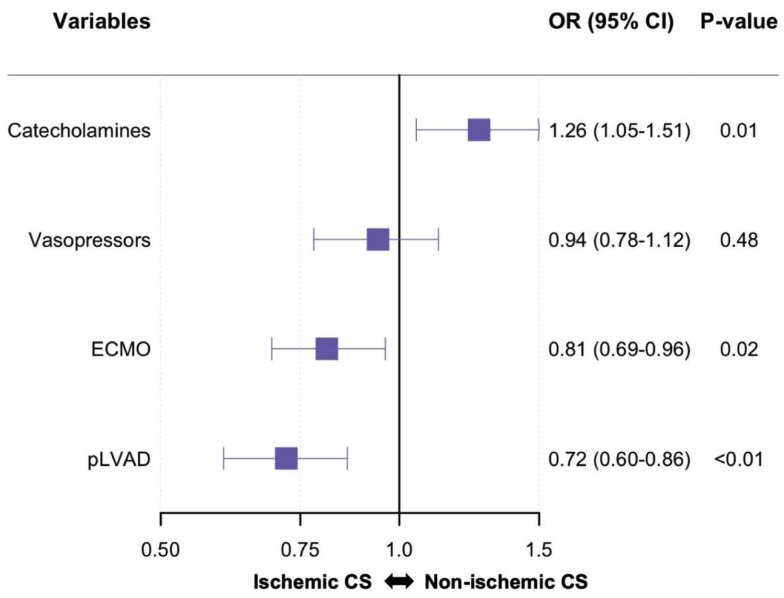
Use of treatments in patients with non-ischemic vs. ischemic cardiogenic shock. Logistic regression model with cause of cardiogenic shock (CS, non-ischemic vs. ischemic) as the dependent variable and all shown baseline treatments as independent variables. Additionally, all models were adjusted for age, sex, prior cardiac arrest, time to return of spontaneous circulation, shock index ≥1, baseline lactate and baseline pH. Odds ratios (OR) are shown on the x-axis. Change in continuous variables was modeled as change per standard deviation. CI: confidence interval; ECMO: veno-arterial extracorporeal membrane oxygenation; pLVAD: perivascular left-ventricular assist device.

In the overall cohort, 764 patients (78.3%) were treated with catecholamines, 701 patients (74.3) were treated with vasopressors, 256 patients (26.2%) were treated with ECMO and 162 patients (16.6%) were treated with pLVAD. After adjustment for relevant confounders, patients with non-ischemic CS were more likely to be treated with catecholamines, but less likely to be treated with ECMO or pLVAD. Use of vasopressors was equally associated with non-ischemic vs. ischemic CS (Figure 2).

### 3.3. Outcome

During a median follow-up of 26 days, 547 deaths (55.9%) occurred in the overall cohort, corresponding to an unadjusted survival probability of 38% (95% confidence interval (CI): 35%–42%). Unadjusted survival probabilities in patients with non-ischemic vs. ischemic CS were 36% (95% CI: 32%–42%) vs. 39% (95% CI: 35%–45%).

**Figure 3:**
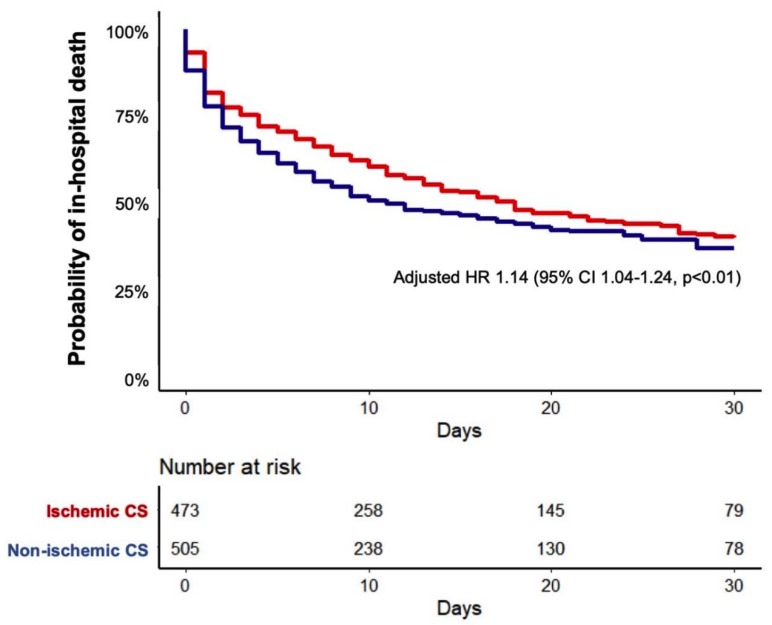
30 day in-hospital mortality in patients with non-ischemic vs. ischemic cardiogenic shock. Multivariable Cox regression model is adjusted for age, sex, prior cardiac arrest, time to return of spontaneous circulation, shock index ≥1, baseline lactate and baseline pH. HR: hazard ratio; CS: cardiogenic shock.

After adjustment for relevant confounders in the Cox regression model, non-ischemic CS was significantly associated with an increased risk of 30-day in-hospital mortality (hazard ratio 1.14, 95% CI: 1.04–1.24, *p* < 0.01; Figure 3).

## 4. Discussion

In this large clinical study, non-ischemic CS differed significantly from ischemic CS in regard to patient characteristics, use of treatments and outcome. Patients with non-ischemic CS were more likely to present in a worse clinical condition (unfavorable hemodynamics and more mechanical ventilation) but were less likely to have cardiovascular risk factors/comorbidities (younger, less likely male, less likely to smoke/have diabetes). Additionally, use of treatment differed significantly between patients with non-ischemic vs. ischemic CS; patients with non-ischemic CS were more likely to be treated with catecholamines, but less likely to be treated with mechanical circulatory support devices. Importantly, non-ischemic CS conveyed a higher risk of 30 day in-hospital mortality, even after adjustment for relevant confounders.

### 4.1. Differences in Patient Characteristics Between Non-Ischemic vs. Ischemic CS

CS itself is defined as a severe hypoperfusion with subsequent end-organ damage caused by an impaired cardiac function [2]. While this is true for the overall CS population, different CS etiologies might also be related to different patient characteristics. In this analysis, patients with non-ischemic CS had distinct characteristics as compared to patients with ischemic CS, including younger age, a lower likelihood of male sex, and a better cardiovascular risk profile, but worse hemodynamic parameters and more mechanical ventilation upon presentation. These findings are partly in line with those of a recent study evaluating CS patients admitted to intensive care units [11]. In this study, patients with non-ischemic CS were also observed to be more frequently younger females with a better cardiovascular risk profile; but had better hemodynamics and less mechanical ventilation than patients with ischemic CS. This might be explained by the different settings (hospital admission vs. intensive care unit admission) and differences in the statistical methods used in the two studies (adjusted logistic regression vs. observed frequencies) [11].

Differences in patient characteristics, even beyond those evaluated in this study, might have relevant implications for diagnosis and management of CS; e.g., the finding that patients with non-ischemic CS were more likely to have worse hemodynamic parameters could indicate that these patients warrant more progressive treatment. However, differences in hemodynamic parameters might also be explained by a different use of vasopressors in both groups (e.g., higher doses in patients with ischemic CS), which was not accounted for in this analysis. In this regard, the recently proposed CS classification of the Society for Cardiovascular Angiography and Interventions might help to identify patients at different CS stages [13]. On top of “classic CS”, “deteriorating CS” and “in extremis CS”, this classification importantly adds definitions for patients “at risk of CS” and with “beginning CS”. Application of this classification in clinical practice might improve early recognition of CS and could guide use of treatments, e.g., earlier initiation in patients with beginning CS or more invasive treatments in patients with more advanced CS [9,10]. Additionally, the utilization of “CS teams” seems to be promising to improve outcomes in non-ischemic CS. Not only is CS a complex disease impacting multiple organs, for which CS teams might foster interdisciplinary care; CS teams could also develop dedicated diagnostic and treatment algorithms, tailored specifically to the underlying etiology [14,15].

### 4.2. Use of Treatments in Patients with Non-Ischemic vs. Ischemic CS

Currently, the only evidence-based treatment for CS is early revascularization of the culprit vessel in patients with ischemic etiology [3,7]. For patients with non-ischemic CS, no intervention has yet shown to improve outcomes in randomized controlled trials [2]. In the presented study, patients with non-ischemic CS were most likely treated with catecholamines. Catecholamines are commonly used in the treatment of CS, as they have a positive inotropic effect which provides support for the failing myocardium. However, they also increase oxygen demand and may thereby negatively impact mortality in this setting [1].

Over the past decades, mechanical circulatory support devices have been increasingly used as an alternative to catecholamines in the treatment of CS [16]. Interestingly, the present analysis indicates that mechanical circulatory support is more likely to be used in patients with ischemic CS. This might relate to the fact that patients with ischemic CS are predominantly treated in the catheterization laboratory. Implantation of ECMO or pLVAD might be more feasible in these patients, as no additional transport is required. In principle, patients with non-ischemic CS might also benefit from mechanical circulatory support. Especially, left-ventricular unloading using a pLVAD seems to be intriguing for treatment of non-ischemic CS, as this device could support the failing myocardium and thereby enhance its chances to recover/stabilize [17]. However, there is currently no randomized evidence which supports the use of ECMO/pLVAD in CS, and observational studies even indicated harm with the use of pLVAD in CS [12,18,19]. As use of mechanical circulatory support is inevitably linked to an increase in complications, such as vascular complications at the access site or bleedings, more research is needed to determine which patients do have a favorable benefit/risk ratio with this approach.

### 4.3. Mortality in Patients with Non-Ischemic vs. Ischemic CS

Lastly, this study indicated a significant and relevant higher mortality risk for patients with non-ischemic vs. ischemic CS. Based on the available evidence on early revascularization of the culprit vessel in ischemic CS, it can be speculated that the higher risk in non-ischemic CS stems from the lack of evidence-based treatments. In general, the main approach to treatment of non-ischemic CS is to support circulatory function and to prevent/minimize end-organ damage until the native heart recovers [2]. In some scenarios, such as reversible myocardial dysfunction due to acute rejection in heart transplant recipients, causative treatments might be available. However, for the majority of non-ischemic CS cases there is no direct treatment [2]. Again, it can only be speculated if the use of pLVAD for left-ventricular unloading would improve outcomes in non-ischemic CS, as the respective evidence is lacking. The high mortality risk observed in this study highlights the need for effective treatments in this CS sub-population and calls for intensified research efforts in this area.

### 4.4. Limitations

The strength of this study is the use of a large CS cohort with available data on important outcome predictors, which allowed for a comparison of non-ischemic vs. ischemic CS with relevant adjustment.

Limitations are inevitably linked to the observational design of our study, which does not make it possible to rule out unmeasured/residual confounding, which might explain the significant differences observed between patients with non-ischemic vs. ischemic CS. Patients with CS were identified from the hospitals claims database via ICD-10 codes, and although patient files were manually reviewed to ensure that CS was the primary diagnosis, misclassification might impact our findings. Non-ischemic CS was defined based on the absence/presence of acute myocardial infarction and, therefore, other definitions might have provided different findings. Finally, our study is based on a cohort from a local hospital and, therefore, generalizability to other settings might be limited.

## 5. Conclusions

In this large cohort of CS patients, non-ischemic CS accounted for more than 50% of all cases and was linked to different patient characteristics as compared to ischemic CS. There were significant and relevant differences the in use of treatments, as patients with non-ischemic CS were more likely to be treated with catecholamines, but less likely to be treated with mechanical circulatory support. Importantly, non-ischemic CS was independently associated with a worse prognosis, even after adjustment for relevant confounders.

The described differences in patient characteristics call for dedicated diagnostic algorithms for patients with non-ischemic vs. ischemic CS and might even influence enrolment criteria for randomized controlled trials. As mortality risk was higher in non-ischemic CS, future research should not only focus on ischemic CS, but evaluate treatment strategies for patients with non-ischemic CS, too. In particular, there is a need for evidence regarding the use of mechanical circulatory supports in non-ischemic CS, as these devices are underused in this setting and as the available evidence stems exclusively from ischemic CS trials.

## Figures and Tables

**Table 1:** Baseline characteristics of the study cohort.

	All (*N* = 978)	Non-Ischemic CS (*N* = 505)	Ischemic CS (*N* = 473)	*p*-Value
**Demographics**				
Age (years)	70.0 (58.0, 79.0)	69.0 (55.0, 78.0)	71.0 (61.0, 79.0)	<0.01
Male No. (%)	683 (69.8)	333 (65.9)	350 (74.0)	<0.01
Female No. (%)	295 (30.2)	172 (34.1)	123 (26.0)	<0.01
**Cardiovascular risk factors**				
Body mass index (kg/m²)	24.7 (24.2, 28.3)	24.8 (23.5, 29.0)	24.7 (24.2, 28.0)	0.65
Smoking No. (%)	295 (30.3)	125 (24.9)	170 (36.2)	<0.01
Arterial hypertension No. (%)	484 (49.8)	224 (44.6)	260 (55.3)	<0.01
Hypercholesterinemia No. (%)	98 (10.1)	52 (10.4)	46 (9.8)	0.85
Diabetes mellitus No. (%)	260 (26.7)	116 (23.1)	144 (30.6)	<0.01
Chronic kidney disease No. (%)	174 (17.9)	106 (21.1)	68 (14.5)	<0.01
Prior myocardial infarction No. (%)	237 (24.4)	132 (26.3)	105 (22.3)	0.17
Prior stroke No. (%)	95 (9.8)	48 (9.6)	47 (10.0)	0.90
**Presentation**				
Prior cardiac arrest No. (%)	567 (58.0)	314 (62.3)	253 (53.5)	<0.01
Time until return of spontaneous circulation	0 (0, 20.0)	2.0 (0, 20.0)	0 (0, 20.0)	0.13
Refractory cardiac arrest No. (%)	116 (28.0)	57 (24.7)	59 (32.2)	0.11
Mechanical ventilation No. (%)	664 (68.2)	357 (71.3)	307 (65.0)	0.04
Ejection fraction				
Preserved No. (%)	134 (17.2)	71 (18.0)	63 (16.3)	0.60
Mid-ranged No. (%)	127 (16.3)	49 (12.4)	78 (20.2)	<0.01
Reduced No. (%)	520 (66.6)	275 (69.6)	245 (63.5)	0.08
**Hemodynamics**				
Systolic blood pressure (mmHg)	104.5 (86.0, 126.0)	100.0 (81.0, 121.0)	110.0 (90.0, 132.0)	<0.01
Diastolic blood pressure (mmHg)	60.0 (47.7, 78.0)	58.0 (45.0, 74.0)	65.0 (50.0, 80.0)	<0.01
Heart rate (bpm)	90.0 (71.0, 110.0)	90.0 (70.0, 114.8)	89.0 (72.0, 106.0)	0.20
Shock index ≥1 No. (%)	307 (35.1)	200 (43.2)	107 (26.0)	<0.01
**Laboratory**				
Lactate (mmol/L)	4.0 (2.0, 8.0)	4.0 (2.0, 9.0)	3.0 (2.0, 8.0)	0.05
pH	7.3 (7.2, 7.4)	7.3 (7.2, 7.4)	7.3 (7.2, 7.4)	0.93
Estimated glomerular filtration rate (mL/min/1.73 m^2^)	42.8 (28.5, 58.9)	38.5 (24.9, 54.7)	47.2 (32.9, 62.8)	<0.01
Aspartate transaminase (U/I)	122.0 (47.0, 381.3)	109.0 (41.0, 352.5)	144.5 (54.0, 430.0)	0.02
Creatine kinase (U/I)	207.5 (101.9, 681.2)	144.0 (81.0, 351.4)	346.5 (149.8, 1230.4)	<0.01
High-sensitive Troponin T (pg/mL)	221.5 (54.9, 1410.0)	106.0 (44.2, 415.7)	592.0 (82.0, 2701.3)	<0.01
C-reactive protein (mg/L)	16.0 (5.0, 72.0)	19.0 (6.0, 72.6)	14.0 (5.0, 71.1)	<0.01

Baseline characteristics of the overall study cohort and stratified by non-ischemic/ischemic cardiogenic shock (CS).
